# Mitochondrial pearling: membrane biophysics, Ca^2+^-dependent regulation, mtDNA nucleoid dynamics, and emerging links to mitochondrial quality control and neurological disorders

**DOI:** 10.3389/fcell.2026.1943282

**Published:** 2026-09-17

**Authors:** Ye Chen, Fajuan Tang, Xihong Li

**Affiliations:** 1 Department of Emergency Medicine, West China Second University Hospital, Sichuan University, Chengdu, China; 2 Key Laboratory of Birth Defects and Related Diseases of Women and Children (Sichuan University), Ministry of Education, Chengdu, China

**Keywords:** Ca^2+^, mitochondrial pearling, mitochondrial transfer, mitophagy, mtDNA nucleoid, neurological disease, sustained pearling phenotype

## Abstract

Mitochondrial dynamics has long been interpreted primarily through fission and fusion, yet tubular mitochondria can also undergo rapid pearling, a phenomenon in which elongated mitochondria reorganize into a beads-on-a-string morphology while retaining a continuous imaged contour. The occurrence and biological relevance of mitochondrial pearling require careful study. The dimensionless tension–bending ratio used to organize these observations is a heuristic analogy to single-membrane tubes, not a validated quantitative model of the mitochondrial double membrane. Evidence does not yet establish a continuous sequence from pearling through coordinated outer- and inner-membrane scission to mitophagy or intercellular mitochondrial transfer; those links are therefore presented as hypotheses and testable predictions. We use the provisional term “candidate disease-associated sustained pearling phenotype” only for within-study events that meet dynamic pearling criteria and show longer duration or delayed/failed reversal relative to appropriately matched controls. No universal duration threshold or validated pearling-defined disease entity currently exists. Event duration, wavelength, un-pearling kinetics, separate outer- and inner-membrane continuity, and the fate of individual pearls should be measured together to determine whether sustained events are incidental, adaptive, or causally involved in disease.

## 1 Introduction: from beads-on-a-string mitochondria to an expanded framework of mitochondrial dynamics

Mitochondria support cellular metabolism and continually remodel their membranes. Most work over the past 3 decades has interpreted those changes through fission and fusion. Drp1 and its receptors drive fission, whereas Mfn1/2 and OPA1 mediate fusion of the outer and inner membranes (Giacomello et al., 2020; Tábara et al., 2025). That model accounts well for network fragmentation, complementation between organelles, exchange of genetic material, and cell-fate control. It is less suited to rapid, periodic shape changes that leave the membrane topologically intact.

Beaded mitochondria were already being described in the early twentieth century. Lewis and Lewis reported a beads-on-a-string appearance in 1915 (Lewis and Lewis, 1915). Decades later, Webster observed focal mitochondrial accumulation and beaded remodeling in axons during Wallerian degeneration (Webster, 1962). Similar appearances have since been noted in Alzheimer’s disease, ischemia–reperfusion, aging neurons, calcium overload, and experiments involving osmotic perturbation (Zhang et al., 2016; D’Alessandro et al., 2025). Without a common physical account, however, authors generally interpreted them as consequences of injury, intermediates before fission, or descriptive features of axonal pathology. Pearling was not yet recognized as a mode of mitochondrial dynamics in its own right.

Recent work has recast these observations. Sturm et al. described mitochondrial pearling as a Rayleigh–Plateau-type instability set by membrane tension and bending elasticity (Sturm et al., 2025). Live-cell experiments by Landoni et al. then showed frequent, reversible pearling under physiological conditions and linked it to regular mtDNA-nucleoid spacing (Landoni et al., 2026). A contemporaneous perspective placed pearling alongside fission and fusion as another dimension of mitochondrial dynamics (van den Ameele and Prudent, 2026). Related instabilities in the axonal plasma membrane, platelet-producing extensions, and other membrane tubes broaden the physical context (Griswold et al., 2025; Léon et al., 2025). Pearling has already entered the vocabulary of mitochondrial dynamics. In this Review, we separate direct observations from neighboring mechanisms and more speculative links, then ask which disease models can discriminate among them.

Across the evidence reviewed here, morphology, membrane topology, and pathway attribution must be evaluated separately. Periodic beading is not synonymous with fragmentation or swelling; continuity of an imaged matrix or outer-boundary contour does not by itself prove that both mitochondrial membranes remain continuous; and a pathway that shares Ca2+ regulation with pearling is not necessarily mediated by pearling. Box 1 defines the required dynamic observations and quantitative readouts.

Box 1 **Criteria for distinguishing mitochondrial pearling from similar morphologies.**

A beaded, granular, or focally enlarged mitochondrion can arise through several processes that look alike in a still image. Reliable classification requires membrane-continuity data, scission status, the order of events, reversibility, and quantitative kinetics. Morphology alone is insufficient.

- Pearling/mitochondrial pearling: A tubular mitochondrion develops periodic or semiperiodic swellings along its long axis while the imaged contour remains continuous. Reversal to a tubule is common and strengthens identification, but reversibility is not part of the definition because an event may undergo downstream scission or clearance before reversal can be observed. Conventional matrix or outer-boundary imaging does not establish simultaneous continuity of the outer mitochondrial membrane (OMM) and inner mitochondrial membrane (IMM); these topological features should be reported separately.
- λ_p_, τ_p_, and un-pearling rate: λ_p_ denotes the characteristic pearling wavelength or interpearl spacing; τ_p_ denotes the duration of an individual pearling event; and the un-pearling rate denotes the rate at which a beaded structure returns to a tubular morphology after Ca^2+^ withdrawal or relief of stress.
- Fission: Fission ends with topological scission of the mitochondrial membranes, producing physically separate fragments. Drp1, endoplasmic reticulum (ER) contact sites, actin, and associated receptors may participate. A short tubule or fragment records only the endpoint. It does not reveal how it formed and, on its own, is not evidence of pearling.
- Fragmentation: This term describes a network outcome in which long mitochondria are replaced by shorter fragments. Increased fission, impaired fusion, or postinjury breakage can all produce it. A fragmented network, by itself, does not demonstrate pearling.
- Swelling: Expansion of matrix volume enlarges a mitochondrion and is often accompanied by osmotic imbalance, mitochondrial permeability transition pore (mPTP) opening, cristae disruption, or loss of membrane potential. A focal swelling may resemble one pearl. Without a periodic spatial pattern, continuity/topology information, and temporal evolution, it should be classified as swelling or indeterminate morphology; failure to reverse alone does not distinguish swelling from pearling that has progressed to another fate.
- Mitochondria-on-a-string (MOAS): A morphological description of beads-on-a-string mitochondria in disease or stress settings. MOAS can motivate further analysis of pearling dynamics, but static morphology alone does not establish that pearling occurred, that reversal failed, or that individual pearls followed an abnormal fate.
- Candidate disease-associated sustained pearling phenotype (provisional): a pearling event dynamically confirmed in a disease or stress model that, within the same study, retains the imaged contour and shows longer duration (τ_p_) or delayed/failed reversal than controls matched for cell type, stimulus, and observation window. No universal τ_p_ threshold is established. OMM and IMM continuity, swelling, rupture, fragmentation, and terminal depolarization must be assessed separately.

### 1.1 Literature search and evidence-assignment framework

**Search strategy.** This narrative Review was updated by searching PubMed from database inception to 12 August 2026 in Title/Abstract fields using three concept blocks: (1) mitochondria* AND (pearling OR beading OR “beads-on-a-string” OR “beads on a string” OR “mitochondria-on-a-string”); (2) mitochondria* AND (pearling OR constriction) AND (calcium OR cristae OR nucleoid* OR mitophagy OR transfer); and (3) “pearling instability” AND (membrane OR vesicle OR tubule). Crossref was used to verify DOI and bibliographic metadata, and reference lists of key direct studies were searched manually.

**Eligibility and evidence assignment.** English-language, peer-reviewed primary studies were prioritized. We included time-resolved mitochondrial imaging studies that could resolve event onset, periodicity, continuity, duration, or reversal; static pearling-like observations when explicitly labeled as morphological evidence; mechanistic studies of Ca^2+^, cristae, nucleoids, fission, mitophagy, or transfer when they informed a testable link; and non-mitochondrial membrane-tube studies only for the underlying physical analogy. We excluded generic fragmentation or swelling without periodic beading or topological information, non-peer-reviewed abstracts, and non-mitochondrial pearl-like structures from biological claims. Reviews and perspectives were used for context rather than as substitutes for primary evidence.

Evidence level was assigned to each claim, not globally to each paper, according to directness of observation and temporal/causal proximity to pearling. Level A required dynamic capture of pearling itself; Level B comprised static or temporally undersampled pearling-like morphology; Level C comprised adjacent mechanisms without direct capture of a pearling event; and Level D comprised extrapolation or prediction. Because the literature is heterogeneous and the purpose is conceptual synthesis rather than effect-size estimation, no meta-analysis or formal risk-of-bias score was performed.

These definitions distinguish states by dynamics and topology rather than single-frame appearance. The table below lists the minimum observation required for each state, features that are insufficient on their own, and recommended measurements. Reversibility is a measurable outcome of pearling, not an absolute definitional requirement.

**Table:** 

State	Minimum evidence	Insufficient on its own	Key measurements
Physiological pearling	Time-resolved periodic or semiperiodic beading with continuity of the imaged contour during the event; reversal supports identification but is not mandatory if downstream scission or clearance intervenes	A single frame; or a matrix-only/outer-boundary contour used as proof that both OMM and IMM remain continuous	λ_p_; τ_p_; un-pearling rate; membrane-potential recovery; OMM and IMM continuity reported separately
Fission	Direct observation of topological membrane scission or complete separation into two fragments	Short tubular or punctate mitochondria alone	Scission event; Drp1/Mff/Fis1; ER contact site
Fragmentation	Increased fragmentation at the network scale	No demonstration of periodic interpearl spacing or reversal	Network length; branching; fragment count
Swelling	Matrix expansion; loss of membrane potential; evidence of mPTP opening or osmotic change	Rounded mitochondria or focal enlargement alone	Matrix volume; Δψm; mPTP; cristae disruption
Candidate disease-associated sustained pearling phenotype (provisional)	Within-study confirmation of pearling and continuity plus longer τ_p_ or delayed/failed reversal relative to matched controls; no universal duration threshold is established	Static MOAS alone; an abnormal adjacent pathway; a single endpoint; or comparison with unmatched controls	τ_p_; recovery half-time; λ_p_; individual-pearl fate; separate OMM/IMM continuity; exclusion of swelling, rupture, and fragmentation

The A–D hierarchy is applied at the claim level. Level A evidence directly records pearling onset and at least the imaged contour continuity, with wavelength (λ_p_), duration (τ_p_), or reversal when available. Level B evidence is static or temporally undersampled MOAS/beads-on-a-string morphology. Level C evidence concerns an adjacent mechanism—such as Ca^2+^ handling, cristae remodeling, mitophagy, Miro1-dependent transport, or lipid homeostasis—without direct capture of pearling. Level D is an extrapolation that requires both continuous imaging and causal perturbation. Only Level A can support claims about pearling kinetics; Levels B–D are described as morphological clues, adjacent mechanisms, or hypotheses, respectively.

## 2 Biophysical basis of mitochondrial pearling

Rayleigh–Plateau instability occurs when surface tension drives a sufficiently long-wavelength disturbance in a liquid column. Classically, perturbations longer than the cylinder circumference grow and eventually divide the column into regularly spaced droplets (Plateau, 1873; Rayleigh, 1878; Bar-Ziv and Moses, 1994; Nelson et al., 1995). A membrane tube is governed by a related but richer energy balance that includes membrane tension, bending rigidity, and tube radius. Work by Bar-Ziv, Moses, Nelson, Seifert, and colleagues showed experimentally and theoretically that lipid-bilayer tubes can settle into a stable pearled configuration (Bar-Ziv and Moses, 1994; Nelson et al., 1995; Goldstein et al., 1996; Bar-Ziv et al., 1998).

A mitochondrion adds several layers of mechanics to this membrane-tube problem. Its outer and inner membranes differ in composition and organization. Cardiolipin, respiratory-chain complexes, adenosine triphosphate (ATP) synthase, and genome-anchoring sites make the effective bending rigidity of the inner membrane unlike that of the outer membrane. Cristae also bear mechanical load; they are internal elastic supports rather than passive folds. The matrix contributes a further variable because it contains Ca^2+^, Mg^2+^, H^+^, phosphate, proteins, and mtDNA nucleoids. Ion movement can therefore translate an osmotic change into a rapid change in membrane tension.

The dimensionless ratio Σ = σR^2^/κ_eff is used here as a heuristic parameter adapted from single-membrane tube models, not as a quantitatively validated constitutive model of mitochondria. In this notation, σ is membrane tension, R is tube radius, and κ_eff is a coarse-grained parameter that summarizes bending resistance, cristae support, and membrane coupling. A critical value has not been measured in living mitochondria, and no unique κ_eff can yet be assigned to the coupled OMM–IMM system. Double-membrane coupling, area–volume constraints, transmembrane pressure differences, spatially varying cristae, and external forces can all shift the instability threshold. The ratio therefore organizes qualitative predictions—higher tensile load or radius favors instability, whereas stronger elastic support opposes it—but should not be used to infer a threshold, bead size, or wavelength quantitatively. Ion flux, lipid remodeling, and cytoskeletal force provide possible routes into this regime (Griswold et al., 2025; Sturm et al., 2025), and their contributions may overlap.

Topological scission separates fission from pearling (Figure 1). Fission ends in physical separation, whereas pearling first produces multiple swellings and necks along the mitochondrial contour. Electron-microscopy studies of canonical fission provide useful structural context: platinum-replica EM shows actin arrays at mitochondrial constrictions, and cryo-electron tomography can resolve mitochondrial membrane morphology (Friedman et al., 2011; Korobova et al., 2013; Cho et al., 2017; Chakrabarti et al., 2018; Yang and Svitkina, 2019; Mageswaran et al., 2023; Sturm et al., 2025; Landoni et al., 2026). Although EM provides high structural resolution, the cited studies did not capture time-resolved pearling, plausibly because such events are dynamic and may be sparse. Cryo-ET nevertheless remains valuable for resolving membrane morphology during captured events, including OMM and IMM continuity, cristae organization, and the distribution of membrane-associated macromolecules. Complementary live-cell imaging is required to distinguish an elongated continuous neck from completed scission and to establish event timing; a matrix or OMM contour alone should not be treated as proof of IMM continuity. Pearling is not simply incomplete fission; the relationship between pearling, neck maturation, and any later scission is an experimental question.

**FIGURE 1:**
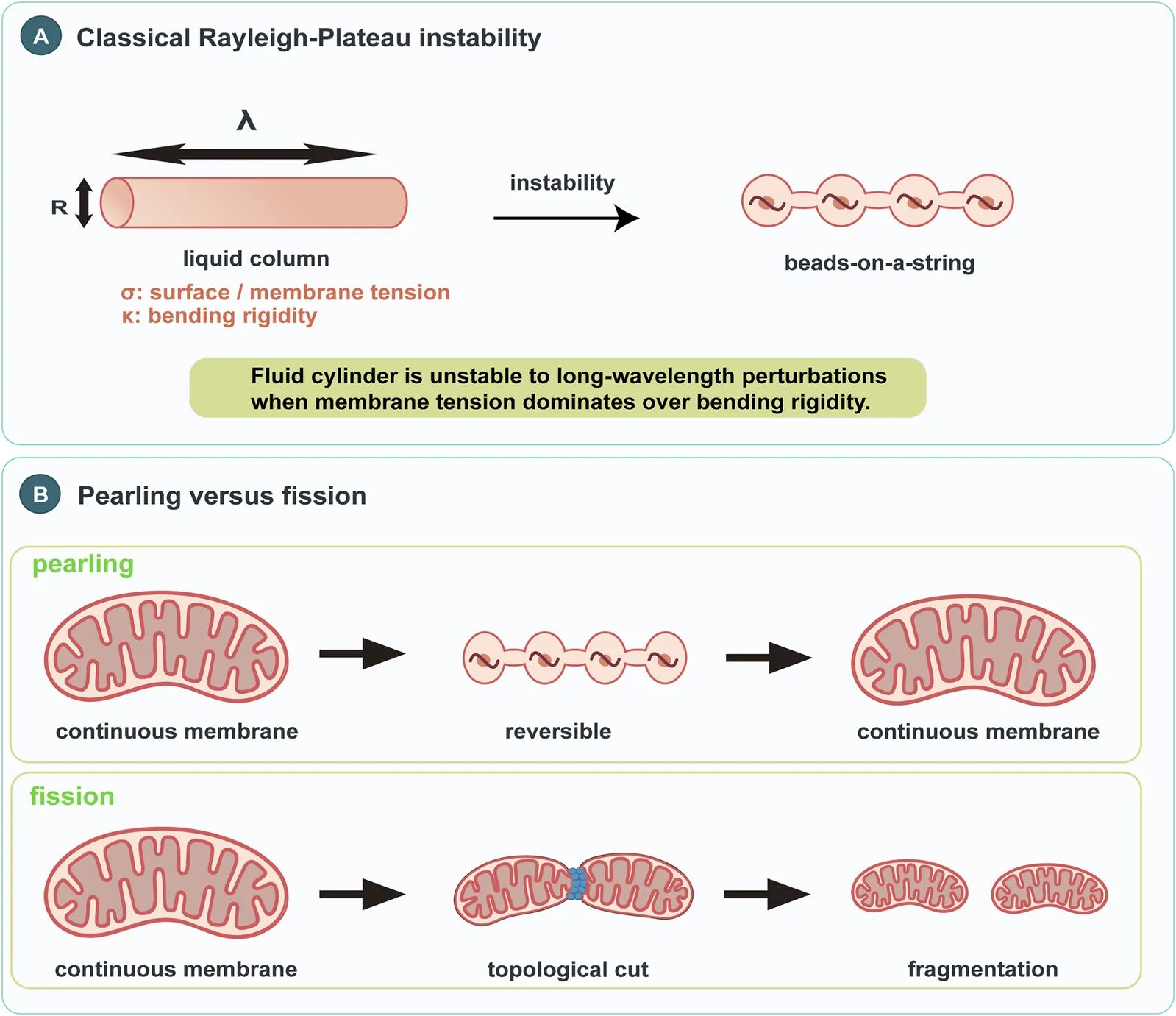
Mitochondrial pearling: biophysics and distinction from fission. **(A)** Rayleigh–Plateau instability provides a qualitative physical analogy for pearling of membrane tubes. Tension (σ), bending resistance (κ), tube radius (R), and wavelength (λ) interact to permit periodic constrictions; the mitochondrial double membrane and cristae add unmodeled complexity. **(B)** Fission ends in topological scission, whereas pearling initially preserves continuity of the imaged contour and is often reversible if no downstream conversion intervenes. The schematic does not establish simultaneous OMM and IMM continuity and is not to scale.

Cristae architecture is expected to influence the pearling threshold, but its effect should be framed as a mechanical hypothesis rather than a measured stiffness law. OPA1 and the mitochondrial contact site and cristae organizing system (MICOS) maintain inner-membrane organization; their disruption is associated with altered cristae, nucleoid organization, and increased susceptibility to beaded morphology (Frezza et al., 2006; Cogliati et al., 2013; Anand et al., 2014; Fry et al., 2024; Sturm et al., 2025). These observations are consistent with reduced or spatially heterogeneous effective support, but they do not yield a calibrated κ_eff or prove that cristae disruption alone causes pearling.

Cristae are distributed nonuniformly along the mitochondrial long axis, so κ_eff is unlikely to be spatially uniform. Local differences in crista density, orientation, and membrane contacts could shift the preferred neck positions and produce deviations in pearl size or spacing. Coupling between the OMM and IMM could also increase the effective resistance to bending relative to either membrane alone, but neither the magnitude of this increase nor a universal coupling factor is known. Measurements of λ_p_ and τ_p_ should therefore be analyzed together with local crista architecture rather than interpreted through a uniform-cylinder model.

## 3 Ca^2+^ as an important trigger of mitochondrial pearling

Ca^2+^ connects mitochondrial morphology, metabolism, and cell fate. Mitochondrial Ca^2+^ handling combines ER–mitochondrial contacts with transport through voltage-dependent anion channels (VDACs), the mitochondrial calcium uniporter (MCU) complex, and the mitochondrial Na^+^/Ca^2+^/Li^+^ exchanger (NCLX). Bulk cytosolic Ca^2+^ is about 100 nM at rest, but local concentrations can rise sharply during receptor activation, synaptic firing, muscle contraction, or stress. At mitochondria-associated membranes (MAMs), the inositol 1,4,5-trisphosphate receptor (IP3R)–glucose-regulated protein 75 (GRP75)–voltage-dependent anion channel 1 (VDAC1)–MCU axis exposes mitochondria to high-Ca^2+^ signals released from the ER across nanometer-scale gaps (Csordás et al., 2010; Rizzuto et al., 2012; Giorgi et al., 2015). Once Ca^2+^ reaches the intermembrane space, MCU/mitochondrial calcium uniporter b (MCUb) conducts it into the matrix, with mitochondrial calcium uptake proteins 1/2/3 (MICU1/2/3) and essential MCU regulator (EMRE) regulating the complex (Baughman et al., 2011; De Stefani et al., 2011; Patron et al., 2014) (Figure 2).

**FIGURE 2:**
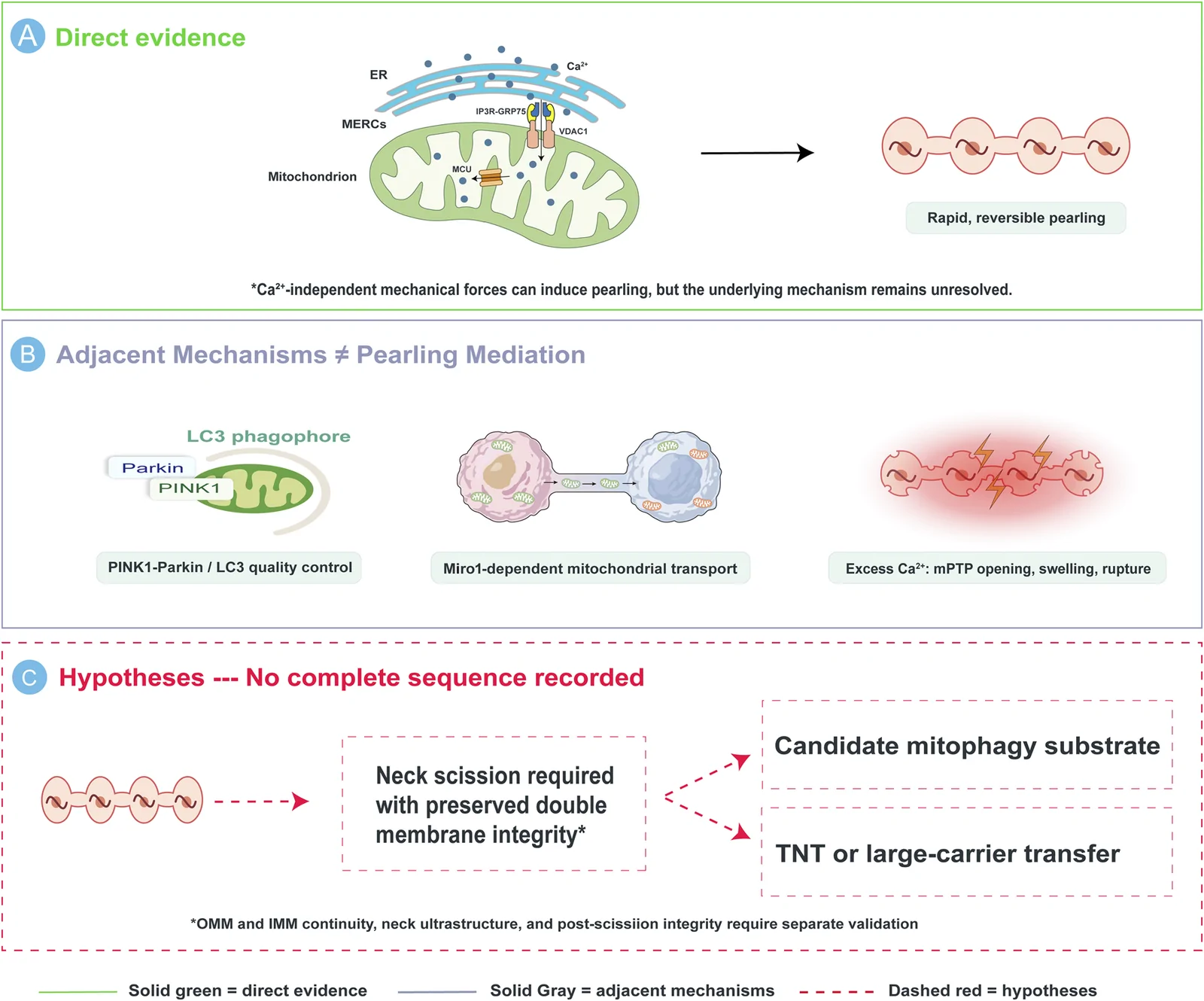
Evidence-stratified framework connecting mitochondrial pearling with Ca^2+^ handling, adjacent pathways, and hypothetical downstream fates. **(A)** Direct evidence. ER-to-mitochondria Ca^2+^ transfer at mitochondria–ER contact sites (MERCs) is illustrated through the IP3R–GRP75–VDAC1 complex, followed by matrix uptake through MCU. Direct time-resolved studies support rapid, reversible pearling after mitochondrial Ca^2+^ loading. Ca^2+^-independent mechanical forces could induce pearling but their nature seems unknown as of now. **(B)** Adjacent mechanisms. PINK1/Parkin–LC3 quality control, Miro1-dependent mitochondrial transport, and excessive-Ca^2+^ injury involving mPTP opening, swelling, and membrane rupture are shown as established or mechanistically adjacent processes. Their shared regulation by Ca^2+^ or mitochondrial stress does not establish mediation by pearling. **(C)** Hypotheses. A continuous pearled tubule could become a candidate mitophagy substrate or a unit for TNT or large-carrier transfer only after neck scission. Such conversion would require preserved double-membrane integrity; OMM and IMM continuity, neck ultrastructure, and post-scission integrity remain to be validated separately. No complete sequence from pearling through scission to mitophagy or carrier-mediated transfer has been recorded. Solid green denotes direct evidence, solid gray denotes adjacent mechanisms, and dashed red denotes hypothetical relationships. The schematic is conceptual and not quantitative. Abbreviations: ER, endoplasmic reticulum; GRP75, glucose-regulated protein 75; IMM, inner mitochondrial membrane; IP3R, inositol 1,4,5-trisphosphate receptor; LC3, microtubule-associated protein 1 light chain 3; MCU, mitochondrial calcium uniporter; MERCs, mitochondria–ER contact sites; Miro1, mitochondrial Rho GTPase 1; mPTP, mitochondrial permeability transition pore; OMM, outer mitochondrial membrane; PINK1, PTEN-induced kinase 1; TNT, tunneling nanotube; VDAC1, voltage-dependent anion channel 1.

A Ca^2+^ signal reaches the pearling machinery through a sequence of physical changes. After cytosolic Ca^2+^ rises, MAM microdomains and the MCU complex promote Ca^2+^ entry into the matrix. The accompanying influx of counterions and water increases matrix osmotic pressure and membrane tension, eventually driving Σ above the pearling threshold. Rapid light-sheet imaging by Landoni et al. showed that Ca^2+^ influx can induce pearling within seconds and that the process can occur independently of the canonical fission machinery (Landoni et al., 2026). This observation provides a more direct physical explanation for many previous reports of mitochondrial constriction during calcium overload.

Ca^2+^-associated pearling should be separated from Ca^2+^-associated injury. Matrix Ca^2+^ entry, counterion movement, and water influx provide a plausible route to a rapid increase in osmotic pressure and membrane tension, and direct imaging supports a class of reversible events after Ca^2+^ influx. By contrast, sustained high-conductance mPTP opening can produce swelling, cristae disorganization, IMM fragmentation, or OMM rupture (Wolf et al., 2017; Bernardi et al., 2022). Such injury is not evidence of sustained pearling unless time-resolved imaging first verifies the pearling pattern and continuity and then shows prolonged τ_p_ or delayed reversal relative to matched controls. Mechanically induced events without a detectable Ca^2+^ increase further show that Ca^2+^ is an important trigger, not a universal requirement.

Recovery from pearling begins with removal of matrix Ca^2+^ and restoration of membrane homeostasis. NCLX extrudes matrix Ca^2+^, although direct evidence that it relaxes membrane tension or speeds un-pearling is still lacking (Palty et al., 2010; Kostic et al., 2015). Transient, low-conductance openings of mPTP could relieve osmotic pressure, whereas persistent high-conductance opening marks irreversible injury (Basso et al., 2005; Bernardi et al., 2022). Return to a tubular network will also depend on cardiolipin maintenance, clearance of lipid peroxides, the balance of long and short OPA1 isoforms, and repair of cristae.

Ca^2+^ also regulates pathways adjacent to pearling. Ca^2+^ oscillations can initiate PINK1–Parkin-associated quality control (Yu et al., 2021), and the EF-hand domains of Miro1 contribute to Ca^2+^-sensitive mitochondrial transport (Ahmad et al., 2014). These findings demonstrate shared upstream Ca^2+^ regulation; they do not show that pearling mediates mitophagy or mitochondrial transfer. Figure 2 therefore depicts these pathways as adjacent evidence rather than as branches of a pearling-controlled fate cascade.

The take-home message is therefore threefold: Ca^2+^ influx can trigger a class of rapid pearling events, and its subsequent withdrawal can accompany their reversal; Ca^2+^ independently regulates downstream quality-control and transport pathways in which a role for pearling remains untested; and Ca^2+^-independent mechanical force can also induce pearling. A single Ca^2+^ dose-fate hierarchy is not established.

## 4 Candidate sustained pearling as a provisional operational framework

Candidate disease-associated sustained pearling is a provisional operational phenotype, not a distinct disease entity or a synonym for MOAS, fragmentation, or swelling. No validated absolute τ_p_ cutoff exists. Classification therefore requires a within-study comparison showing that a dynamically confirmed pearling event lasts longer or reverses more slowly than in controls matched for cell type, stimulus, and imaging window, while alternative outcomes are excluded. To our knowledge, no neurological disease model currently satisfies this full set of criteria; current disease associations should be treated as candidates for validation.

## 5 Direct functional evidence: pearling and mtDNA nucleoid redistribution

Mammalian cells contain numerous copies of mtDNA. These genomes are not freely dispersed but are packaged into nucleoid structures approximately 100 nm in size. Super-resolution imaging shows that most nucleoids contain one or a few mtDNA copies and are closely associated with the inner membrane and cristae (Garrido et al., 2003; Brown et al., 2011; Kukat et al., 2011; 2015; Bogenhagen, 2012). In healthy cells, nucleoids often exhibit a semiregular distribution, with neighboring nucleoids commonly separated by 1–2 μm. Random diffusion alone cannot readily explain this spatial precision.

Before the work of Landoni et al., three main models were used to explain nucleoid distribution: coupling of mtDNA synthesis and fission at ER–mitochondrial contact sites (Lewis et al., 2016), transport of nucleoids along the inner membrane regulated by ATPase family AAA domain-containing protein 3A (ATAD3A) (Zhao et al., 2019; Ishihara et al., 2022), and ER-mediated dynamic stretching of mitochondria that promotes long-range nucleoid movement (Qin et al., 2020). These models account for transport, replication, and partitioning but do not fully explain why steady-state spacing has a relatively stable characteristic length. Pearling offers a geometric explanation for this feature.

Landoni et al. showed that the characteristic wavelength of physiological pearling is approximately 1 μm, closely matching the spacing between nucleoids (Landoni et al., 2026). A single pearling event can displace nucleoids within a tubule toward the centers of adjacent pearls. After un-pearling restores a tubular morphology, the nucleoids can remain at their new positions. Repeated brief pearling events could therefore maintain nucleoid distribution in a dynamic steady state. In other words, nucleoid spacing may not be a purely static structural property but a pattern continuously reset by Ca^2+^–pearling cycles.

Cristae and OPA1 status connect pearling mechanics to mtDNA organization, but the relationship is not monotonic. Reduced OPA1 or MICOS function can weaken or spatially disorder inner-membrane support and is associated with altered pearling and nucleoid spacing (Fry et al., 2024; Sturm et al., 2025; Landoni et al., 2026). The same crista disruption that lowers a local mechanical threshold may also remove the relatively uniform support and membrane landmarks needed for regular bead spacing and reproducible nucleoid repositioning. More frequent or easier beading therefore does not predict more organized nucleoids. Patient-derived OPA1-associated autosomal dominant optic atrophy cells show disrupted cristae and nucleoid organization (Macuada et al., 2024), but dynamic τ_p_, local crista architecture, and un-pearling still need to be measured together.

Direct evidence currently ends with pearling-associated nucleoid redistribution. Proposals that a pearl subsequently isolates a damaged nucleoid for focal fission, nucleoid-phagy, or mitophagy remain hypotheses (Liu et al., 2024b; Newman et al., 2024). Existing nucleoid-clearance studies concern cytosol-exposed mtDNA–TFAM complexes or other damage contexts, not recognition of a nucleoid enclosed within an intact pearl. Likewise, cGAS–STING activation requires mtDNA access to the cytosol; no continuous sequence from pearling to membrane breach, mtDNA release, and cGAS–STING activation has been shown. These downstream links should be tested rather than inferred from spatial compatibility.

## 6 Pearling before scission-dependent downstream processes: evidence and predictions

Pearling generates a patterned but still connected tubule. Mitophagic engulfment or intercellular transfer of an individual pearl would require at least one additional event: coordinated topological scission that preserves the relevant membrane architecture. No study has followed the same mitochondrion through a complete pearling→neck scission→mitophagy or pearling→neck scission→carrier loading→recipient uptake sequence. The following subsections therefore separate established downstream biology from pearling-specific predictions.

Established mitophagy studies show that depolarized mitochondrial material can recruit PINK1/Parkin or receptor-mediated machinery and be engulfed after network remodeling (Narendra et al., 2008; 2010; Twig et al., 2008; Geisler et al., 2010; Lazarou et al., 2015; Pickrell and Youle, 2015; McWilliams et al., 2016). Reports that mitochondrial division during mitophagy can occur without Drp1 (Yamashita et al., 2016; Burman et al., 2017) broaden the possible scission mechanisms but do not identify pearling as an upstream step. Size compatibility between pearls and autophagic substrates is therefore insufficient evidence of a causal interface.

Structural studies provide contextual, not pearling-specific, evidence. *In situ* cryo-electron tomography has visualized spherical mitochondrial fragments and phagophore engulfment after depolarization (Rose et al., 2025), but those snapshots did not establish a preceding pearling event or continuity at pearl necks. The decisive experiment remains continuous live imaging followed by correlative ultrastructure on the same mitochondrial segment.

Hypothesis and prediction: if pearling contributes to mitophagy, it should reproducibly precede local loss of membrane potential, coordinated OMM/IMM scission, recruitment of PINK1/Parkin or receptor-mediated machinery, LC3-positive engulfment, and lysosomal delivery on the same mitochondrial segment. Selective suppression or enhancement of pearling should then change this sequence without simply altering Ca^2+^ load, global fission, or mitochondrial injury. Until these criteria are met, pearling and mitophagy should be treated as potentially parallel responses.

Mitochondria-derived vesicles (MDVs) should not be placed on a simple size continuum with pearls. Canonical MDVs are generally substantially smaller than a ∼1-μm pearl and can contain one or two mitochondrial membranes, depending on cargo and route (Soubannier et al., 2012). A pearl remains part of a connected double-membrane organelle until proven otherwise. A pearling-to-MDV model would therefore require direct demonstration of neck remodeling, membrane provenance, coordinated or selective OMM/IMM scission, and vesicle trafficking; none is currently available.

### 6.1 Intercellular mitochondrial transfer as a lower-evidence hypothesis

Intercellular mitochondrial transfer can occur through tunneling nanotubes (TNTs), extracellular vesicles, migrasomes, or localized cell fusion (Rustom et al., 2004; Spees et al., 2006; Islam et al., 2012; Pasquier et al., 2013; Phinney et al., 2015; Hayakawa et al., 2016; Torralba et al., 2016; Dong et al., 2017; Jiao et al., 2021; Saha et al., 2022; Borcherding and Brestoff, 2023; Guan et al., 2024; Liu et al., 2024a; Palese et al., 2025). Transfer has been observed from mesenchymal stem cells to injured epithelial cells, from astrocytes to neurons, and in tumor–immune systems (Islam et al., 2012; Phinney et al., 2015; Hayakawa et al., 2016; Saha et al., 2022; Baldwin et al., 2024). These routes differ in cargo capacity, membrane architecture, and biogenesis. Their existence does not demonstrate a shared loading process or a requirement for pearling.

Pearling alone does not create an independently transportable unit because adjacent pearls remain connected. Transfer of a pearl-sized unit would require coordinated neck scission that preserves OMM and IMM integrity, followed by loading into a carrier capable of accommodating an intact mitochondrion. We do not assign ESCRT proteins as candidate executors of this step because current evidence does not place them at the IMM or demonstrate coordinated OMM/IMM scission during pearling. The mechanism and membrane topology are open questions.

No study has continuously recorded pearling, neck scission, carrier loading, and recipient uptake. A discriminating experiment would perturb pearling while preserving global mitochondrial transport and network integrity, then use continuous donor–recipient imaging and correlative ultrastructure to determine whether intact double-membrane units enter TNTs or large mitochondria-containing carriers. A temporal association alone would be insufficient; selective inhibition and rescue are required.

Miro1 is an adjacent transport regulator, not evidence of a pearling-dependent loading step. Manipulating Miro1 alters mitochondrial transfer from mesenchymal stem cells and recipient-cell rescue (Wang et al., 2011; Ahmad et al., 2014), but these studies did not record pearling. Simultaneous imaging of Miro1, donor mitochondrial motion, pearling, neck topology, and recipient uptake is needed before Miro1 can be placed in a pearling-to-transfer sequence.

## 7 Candidate disease-associated sustained pearling: priority models and evidentiary boundaries

Beads-on-a-string mitochondria and abnormalities in pearling-related pathways occur in neurological disease, but similar morphology does not imply a shared mechanism. To our knowledge, no neurological disease model has yet demonstrated the full within-study sequence required for a candidate sustained phenotype: dynamic pearling, continuity of the imaged contour, longer τ_p_ or delayed/failed reversal relative to matched controls, and exclusion of swelling, rupture, and fragmentation. Disease associations are therefore presented as priorities for testing rather than as established examples. Table 1 summarizes candidate disease contexts for mitochondrial pearling and details their mechanistic relevance, current supporting evidence, and key validation needs. Instead, it reports only the current evidence type, why each context is informative, and the minimum event-level validation. Unlisted criteria should not be interpreted as satisfied.

**TABLE 1:** Candidate neurological contexts and minimum validation for sustained pearling.

Candidate context	Current evidence (not pearling confirmation)	Why the context is informative	Minimum event-level validation and boundary
Alzheimer’s disease (AD)	MOAS or beads-on-a-string morphology and Aβ/tau, MAM, and mitochondrial Ca^2+^ abnormalities provide adjacent morphological and mechanistic evidence	Tests whether a static disease morphology maps to a reversible or sustained dynamic event	Acquire live-cell λ_p_, τ_p_, un-pearling, and separate OMM/IMM continuity in matched neurons; exclude swelling, rupture, and fragmentation. MOAS alone is not confirmation (Du et al., 2008; Wang et al., 2009; Calì et al., 2012; Zhang et al., 2016; Fang et al., 2019; Calvo-Rodriguez et al., 2020)
Parkinson’s disease (PD)	PINK1/Parkin quality-control defects and α-synuclein-related MAM and Ca^2+^ abnormalities provide adjacent mechanistic evidence	Tests whether quality-control recruitment follows a dynamically verified pearling event	Track the same mitochondrion from pearling through scission and PINK1/Parkin or LC3 recruitment, with perturbation and rescue. Adjacent pathway abnormalities are not confirmation (Calì et al., 2012; Lazarou et al., 2015; Pickrell and Youle, 2015; Palese et al., 2025)
Huntington’s disease (HD)	Altered mPTP regulation and mitochondrial Ca^2+^ sensitivity provide adjacent mechanistic evidence	Tests whether an altered injury threshold delays reversal or instead causes swelling	Measure τ_p_ and un-pearling together with Δψm, matrix volume, membrane continuity, and mPTP state; distinguish pearling from osmotic expansion (Quintanilla et al., 2013; Gu et al., 2024)
Amyotrophic lateral sclerosis (ALS)	Mitochondrial dysfunction, oxidative stress, and impaired axonal transport define a vulnerable disease context but do not demonstrate pearling	Tests whether long-axon vulnerability selectively changes recovery kinetics	Use continuous axonal imaging with matched stress and prespecified event criteria. Mitochondrial vulnerability alone does not establish sustained pearling (Smith et al., 2019; Rizea et al., 2024)
CMT2A/MFN2-associated axonopathy	MFN2 mutations alter morphology, bioenergetics, and axonal transport; mitofusin activation modifies some phenotypes	Provides a genetic entry point to test transport and fusion effects on recovery without equating fusion with un-pearling	Measure λ_p_, τ_p_, un-pearling, and topology in patient-derived neurons, with MFN2 perturbation and rescue. This is a testing context, not a confirmed example (Franco et al., 2022; Zanfardino et al., 2023)
OPA1/ADOA and cristae pathology	OPA1-dependent crista remodeling and abnormal nucleoid distribution may alter effective mechanical support	Provides a genetic entry point for testing crista-dependent pearling thresholds	Establish temporality between crista change and event kinetics in patient cells; measure OMM/IMM continuity and use OPA1 or OMA1 perturbation and rescue (Frezza et al., 2006; Cogliati et al., 2013; Anand et al., 2014; Fry et al., 2024; Macuada et al., 2024)
Wallerian-like axonal degeneration	Early axonal mitochondrial beading and SARM1-linked NAD^+^/NMN dysregulation provide adjacent morphological and mechanistic evidence	Acute onset permits separation of early beading from downstream axonal disintegration	Continuously image onset, reversal, membrane topology, and axonal integrity during SARM1 perturbation. Historical beading alone is not dynamic pearling evidence (Webster, 1962 ; Osterloh et al., 2012 ; Salvadores et al., 2017 ; Figley et al., 2021 ; Ulshöfer et al., 2022)
Ischemia–reperfusion/stroke	Acute Ca^2+^ and reactive-oxygen-species loads, OPA1 cleavage, mPTP opening, and poststroke transfer provide adjacent mechanisms.	Tests the transition from a reversible event to swelling, rupture, or fragmentation	Image before and after stress withdrawal with λ_p_, τ_p_, Δψm, matrix volume, and OMM/IMM continuity; exclude late injury morphology (Hayakawa et al., 2016; Bernardi et al., 2022; Fry et al., 2024)
Aging	Age-related drift in Ca^2+^ homeostasis, OPA1, mitophagy, Miro1/TNT function, and lipid homeostasis defines a broad context only	Tests whether cumulative homeostatic decline selectively delays reversal.	Compare age-stratified matched cohorts using the same stimulus, imaging window, and event annotation; do not treat aging as a single trigger (Sun et al., 2015 ; Sun et al., 2016 ; López-Otín et al., 2023)

The A–D evidence hierarchy delimits the claims that can be made. Level A can support event-level statements about pearling; Levels B and C identify morphological or mechanistic entry points; Level D propositions offer testable predictions. The central disease-focused question is not whether multiple disorders share one pearling mechanism, but whether any selected model first satisfies the operational criteria and then yields a causal chain to dysfunction.

The highest-priority models should offer a clear genetic or acute-injury entry point and permit single-mitochondrion time-series imaging. First, OPA1/ADOA provides a direct mechanistic entry point linking cristae support, κ_eff, nucleoid distribution, and the pearling threshold (Frezza et al., 2006; Cogliati et al., 2013; Anand et al., 2014; Fry et al., 2024; Macuada et al., 2024). If patient-derived cells or induced pluripotent stem cell (iPSC)-derived neurons show longer τ_p_, altered λ_p_, and failed reversal, and these phenotypes can be corrected by manipulating the OPA1–OMA1 zinc metallopeptidase (OMA1) axis or stabilizing cristae, the model would gain strong support. Second, Charcot–Marie–Tooth disease type 2A (CMT2A)/mitofusin 2 (MFN2) is suitable for testing whether network morphology and axonal transport are coupled to recovery by un-pearling. Fusion and un-pearling are topologically distinct processes, so MFN2 deficiency cannot be assumed to cause failed reversal. MFN2 mutations affect mitochondrial morphology, bioenergetics, and axonal transport, and small-molecule mitofusin activators provide tools for causal intervention (Franco et al., 2022; Zanfardino et al., 2023). Third, Wallerian-like axonal degeneration and ischemia–reperfusion models provide an acute time axis that can distinguish brief pearling triggered by Ca^2+^/reactive oxygen species (ROS) from failed reversal, swelling, and downstream cell death (Webster, 1962; Osterloh et al., 2012; Salvadores et al., 2017; Figley et al., 2021; Bernardi et al., 2022; Ulshöfer et al., 2022; Fry et al., 2024).

Alzheimer’s disease (AD), Parkinson’s disease (PD), Huntington’s disease (HD), amyotrophic lateral sclerosis (ALS), and aging remain important, but current evidence makes them more appropriate as adjacent mechanistic contexts than as established examples of disease-associated sustained pearling. In AD, amyloid-β, tau, altered MAM signaling, and mitochondrial Ca^2+^ overload all disrupt mitochondrial homeostasis. MOAS is a useful morphological clue, but classification as sustained pearling still requires dynamic evidence of membrane continuity, prolonged τ_p_, and failed reversal (Du et al., 2008; Wang et al., 2009; Calì et al., 2012; Zhang et al., 2016; Fang et al., 2019; Calvo-Rodriguez et al., 2020). PD offers a different mechanistic context, centered on PINK1/Parkin dysfunction, α-synuclein, and intercellular spread. Per-pearl imaging is needed to determine whether PINK1/Parkin, LC3, or Miro1 influences the fate of an individual pearl (Calì et al., 2012; Lazarou et al., 2015; Pickrell and Youle, 2015; Palese et al., 2025). In HD, increased mPTP susceptibility could prolong osmotic and membrane stress after a pearling trigger; in ALS, disrupted axonal transport and organelle quality control could impede restoration or clearance of affected mitochondria; and during aging, cumulative redox, proteostatic, and bioenergetic decline could weaken the homeostatic recovery required for un-pearling. These are distinct upstream routes by which un-pearling capacity might be reduced, but each remains a testable hypothesis until event-resolved pearling is measured in the relevant model (Quintanilla et al., 2013; Sun et al., 2015; Sun et al., 2016; Smith et al., 2019; López-Otín et al., 2023; Gu et al., 2024; Rizea et al., 2024).

From a therapeutic perspective, the objective should not be to abolish pearling itself. Physiological pearling may support mtDNA distribution and focal quality control; abnormal duration, frequency, and fate divergence are the more relevant targets. At present, mPTP modulators, mitophagy enhancers, regulators of the ubiquitin-specific peptidase 30 (USP30)/PINK1 axis, allosteric MFN2 activators, and OPA1/OMA1 modulators are therefore better regarded as tools for testing disease-associated sustained pearling readouts or as candidate interventions, not as established treatments that specifically target sustained pearling (Andreux et al., 2019; Fang et al., 2019; Franco et al., 2022; Antico et al., 2025; Kazi et al., 2025). The working model can advance from a correlated phenotype to a classifiable and actionable pathological mechanism only if selective alteration of pearling—while controlling for concurrent changes in Ca^2+^, membrane potential, swelling, and fission—demonstrates causal relationships of τ_p_, λ_p_, and un-pearling rate with membrane-potential stability, respiration, the precision of mtDNA distribution, neurite integrity, and cell survival, and if rescuing pearling dynamics restores functional outcomes.

## 8 Measurement challenges, validation strategies, and scope of mitochondrial pearling

The next step in pearling research is not to accumulate further descriptions of static morphology but to establish reproducible dynamic readouts and use them to test links between pearling and nucleoid distribution, quality control, intercellular transfer, and disease phenotypes. Three connected questions must be addressed: How can individual pearling events be captured reliably? Which mechanistic links have direct support? Under what conditions should current interpretations be restricted or rejected?

### 8.1 Methodological bottlenecks and measurement framework

The first major limitation is the mismatch between the spatial scale of pearl necks and nucleoids and the speed of onset and reversal. A tiered workflow is appropriate: rapid multi-channel light-sheet imaging for morphology, matrix Ca^2+^, and membrane potential; targeted super-resolution imaging for necks and nucleoids; and event-triggered correlative cryo-CLEM, cryo-FIB-SEM, or cryo-ET for ultrastructure. Matrix, OMM, and IMM reporters should be interpreted separately because a continuous matrix or OMM contour does not prove that both membranes are continuous. Event-triggered cryo-ET can complement and validate these fluorescence-based continuity assignments by directly resolving the OMM, IMM, and cristae at the captured event site. Correlative localization should link the tiers, and phototoxicity, photobleaching, probe overexpression, and missed-event rates should be prespecified quality-control measures (Chen et al., 2014; Mahamid et al., 2016; Schermelleh et al., 2019; Gwosch et al., 2020).

The second bottleneck is the lack of direct measurements of physical parameters. In addition to interpearl spacing, duration, frequency, and un-pearling rate, studies should separately record the osmotic pressure difference ΔΠ and hydrostatic pressure difference ΔP and estimate the effective mechanical properties of the mitochondrial inner membrane. The fluorescence lifetime of Flipper-type probes can provide an indirect readout of membrane-lipid packing and changes in tension, but their use in the inner mitochondrial membrane requires calibration for subcompartment targeting, lipid composition, and curvature dependence. Combining these measurements with matrix Ca^2+^, membrane potential, and artificial-intelligence-assisted morphological tracking could produce a state map for individual mitochondria. Open standards for event annotation should also record onset, peak, end of reversal, number of pearls, neck radius, membrane continuity, positions of neighboring nucleoids, and the fate of each pearl (Colom et al., 2018; Carpenter et al., 2006; Caicedo et al., 2017; Stringer et al., 2021).

A third question is whether un-pearling is driven solely by restoration of ionic and mechanical parameters or also requires active network regulation. OPA1/OMA1, MICOS, ATAD3A, cardiolipin metabolism, MCU/MICU, NCLX, and mPTP are candidates, but each requires independent mechanistic rationale and direct validation. CRISPR screens could use post-withdrawal τ_p_, the decay rate of a normalized pearling index, the proportion of failed reversals, and membrane continuity as joint primary readouts. Perturbations that produce a reproducible pearling phenotype should then undergo secondary measurements of membrane potential, nucleoid distribution, and single-pearl fate. USP30 is a tractable candidate because its deubiquitinase activity opposes PINK1/Parkin-dependent mitophagy, allowing tests of whether quality-control signaling alters pearling reversal independently of downstream clearance. PHB1/2 should be tested separately as inner-membrane scaffolds that may influence cristae organization and effective membrane support; OPA1 processing and cristae architecture should be measured alongside pearling dynamics. By contrast, the proposed SLP2-cardiolipin microdomain remains a hypothesis because direct localization and perturbation data linking it to pearl-neck formation or reversal are currently lacking. The proposed SLP2–cardiolipin microdomain, in contrast, still lacks direct supporting evidence (Kazi et al., 2025; Rose et al., 2025).

### 8.2 Evidence base and validation sequence for core propositions on mitochondrial pearling mechanisms

Table 2 places core propositions regarding mitochondrial pearling mechanisms alongside their current claim status, unresolved gaps, and the experiments most likely to distinguish among competing explanations. Candidate sustained pearling should be tested first in models with a clear causal entry point, compatibility with continuous imaging, and opportunities for mechanistic rescue.

**TABLE 2:** Evidence base, claim status, and validation sequence for the core propositions.

Core proposition	Current evidence level	Principal gap	Next experiment
Ca^2+^ can trigger rapid, reversible mitochondrial pearling	A (direct dynamic evidence in selected systems)	Generality across cell types, Ca^2+^ loads, and cristae states remains unresolved	Simultaneously measure matrix Ca^2+^, membrane potential, λ_p_, τ_p_, and un-pearling rate while manipulating MCU, NCLX, and mPTP
Rapid pearling can redistribute mtDNA nucleoids and influence spacing in directly imaged models	A (model-specific direct evidence)	Existing evidence is concentrated in specific models; validation is needed in neurons and patient-derived cells	Track retention of nucleoid positions after individual pearling events during OPA1, ATAD3A, or MICOS manipulation and in patient-derived cells
Pearling may create geometry that precedes scission-dependent mitophagy	C for adjacent mitophagy mechanisms; D for the pearling link	No same-mitochondrion sequence from pearling through scission, PINK1/Parkin or receptor recruitment, engulfment, and lysosomal delivery has been demonstrated	Continuously image morphology, OMM/IMM continuity, membrane potential, scission, PINK1/Parkin or receptor recruitment, LC3, and lysosomal delivery on the same mitochondrial segment
After coordinated OMM/IMM scission, a pearl could hypothetically become a unit for TNT or large-carrier transfer	C for adjacent transfer mechanisms; D for the pearling link	No continuous pearling→neck scission→carrier loading→recipient uptake sequence has been recorded; scale matching is insufficient	In MSC–epithelial, astrocyte–neuron, and tumor–immune cocultures, continuously track pearl-neck scission, double-membrane integrity, entry of units into TNTs or large mitochondria-containing vesicles, and arrival in recipient cells
A candidate disease-associated sustained pearling phenotype may exist in selected neurological models	B/C; no disease model currently satisfies the full dynamic criteria	No validated absolute τ_p_ threshold exists, and most disease evidence is static or mechanistically adjacent	Use within-study matched controls and prespecified event-level criteria before testing causality or generalizing across diseases

OPA1/ADOA, CMT2A, and acute axonal injury or ischemia can serve as priority models for determining whether longer pearling duration (τ_p_) or failed reversal is reproducible and whether these changes can be corrected by targeted interventions. Only after reproducible event-level evidence is obtained should the scope expand to complex neurodegenerative diseases and population genetics. Population-scale associations can nominate disease settings worth studying, but they cannot verify a pearling event or establish its causal role.

Pearling is also found outside mitochondria. Related transitions occur in the axonal plasma membrane, platelet-producing extensions, endosomal tubules, and reconstituted membrane tubes (Bar-Ziv and Moses, 1994; Nelson et al., 1995; Gao et al., 2018; Griswold et al., 2025; Léon et al., 2025). Comparisons across these systems can test whether λ_p_ scales with tube radius, how internal supports shift the instability threshold, and whether Ca^2+^, tension, and lipid composition act as a recurring control set. Pearling across these systems, including mitochondrial pearling, therefore provides a clear interface between cell biology and soft-matter physics.

### 8.3 Boundaries of applicability for disease-associated sustained pearling

Whether disease-associated sustained pearling becomes an explanatory disease mechanism depends on the reproducibility of its dynamics, spatial scale, and functional consequences across independent models. The following results would directly limit the scope of the interpretation.

First, disease-associated differences are not reproducible. If preregistered, adequately powered, and appropriately matched studies fail to identify reproducible and practically meaningful between-group differences in pearling frequency, τ_p_, or un-pearling, sustained pearling should not be generalized as a shared mechanism across diseases.

Second, proposed pearl-level fates are not spatially resolved. If PINK1/Parkin, LC3, Miro1, scission, or carrier loading does not reproducibly localize to specified pearls or necks before the outcome occurs, fate should not be assigned to an individual pearl. The more conservative interpretation would be that pearling and the molecular pathway are parallel, tubule-wide responses.

Third, pearling scale is not stably related to nucleoid spacing. If the relationship between characteristic wavelength (λ_p_) and nucleoid spacing cannot be reproduced across cell types, cristae states, or patient-derived models, the interpretation of pearling as a geometric ruler for nucleoids should be restricted to experimental systems with direct supporting evidence.

Fourth, pearling follows irreversible injury. If imaging with sufficient temporal resolution shows that mPTP opening, mitochondrial swelling, or membrane rupture usually precedes pearling, then beads-on-a-string morphologies in acute injury are more likely manifestations of terminal damage than consequences of sustained reversible pearling or failed un-pearling.

Fifth, altering pearling does not affect downstream function. If selective manipulation of pearling, after controlling for Ca^2+^ load, membrane potential, swelling, and overall fission and fusion, does not affect nucleoid distribution, mitophagy, intercellular mitochondrial transfer, or functional outcomes, the proposed causal relationship lacks support. Its scope should then be narrowed or, if necessary, the interpretation abandoned.

## 9 Discussion

Current evidence identifies mitochondrial pearling as a rapid membrane transition influenced by tension, bending resistance, matrix osmotic pressure, Ca^2+^ signaling, and external force, with direct evidence for mtDNA nucleoid redistribution in selected systems. The single-membrane instability ratio is a heuristic, and simultaneous OMM/IMM topology at pearl necks remains unresolved. Continuous imaging and causal perturbation are still lacking for pearling-dependent mitophagy or intercellular transfer; these links require an intervening scission step and are presented as hypotheses. Disease-associated sustained pearling is likewise a provisional within-study phenotype with no validated absolute τ_p_ threshold and no currently established neurological example. Future studies should first capture pearling, membrane topology, and reversal in the same time series, then test whether selective manipulation of pearling changes nucleoid organization or a downstream scission-dependent sequence without confounding Ca^2+^ load, swelling, or global fission. This evidence-stratified framework preserves pearling as a distinct dynamic process while preventing adjacent pathways and static disease morphologies from being overinterpreted as direct consequences.

## References

[B1] Ahmad T. Mukherjee S. Pattnaik B. Kumar M. Singh S. Kumar M. (2014). Miro1 regulates intercellular mitochondrial transport and enhances mesenchymal stem cell rescue efficacy. EMBO J. 33, 994–1010. 10.1002/embj.201386030 24431222 PMC4193933

[B2] Anand R. Wai T. Baker M. Kladt N. Schauss A. C. Rugarli E. (2014). The i-AAA protease YME1L and OMA1 cleave OPA1 to balance mitochondrial fusion and fission. J. Cell Biol. 204, 919–929. 10.1083/jcb.201308006 24616225 PMC3998800

[B3] Andreux P. Blanco-Bose W. Ryu D. Burdet F. Ibberson M. Aebischer P. (2019). The mitophagy activator urolithin A is safe and induces a molecular signature of improved mitochondrial and cellular health in humans. Nat. Metab. 1, 595–603. 10.1038/s42255-019-0073-4 32694802

[B4] Antico O. Thompson P. Hertz N. Muqit M. Parton L. (2025). Targeting mitophagy in neurodegenerative diseases. Nat. Rev. Drug Discov. 24, 276–299. 10.1038/s41573-024-01105-0 39809929

[B5] Baldwin J. Heuser-Loy C. Saha T. Schelker R. C. Slavkovic-Lukic D. Strieder N. (2024). Intercellular nanotube-mediated mitochondrial transfer enhances T cell metabolic fitness and antitumor efficacy. Cell 187, 6614–6630. 10.1016/j.cell.2024.08.029 39276774 PMC11623344

[B6] Bar-Ziv R. Moses E. (1994). Instability and “pearling” states produced in tubular membranes by competition of curvature and tension. Phys. Rev. Lett. 73, 1392–1395. 10.1103/PhysRevLett.73.1392 10056781

[B7] Bar-Ziv R. Moses E. Nelson P. (1998). Dynamic excitations in membranes induced by optical tweezers. Biophys. J. 75, 294–320. 10.1016/S0006-3495(98)77515-0 9649388 PMC1299700

[B8] Basso E. Fante L. Fowlkes J. Petronilli V. Forte M. Bernardi P. (2005). Properties of the permeability transition pore in mitochondria devoid of cyclophilin D. J. Biol. Chem. 280, 18558–18561. 10.1074/jbc.C500089200 15792954

[B9] Baughman J. Perocchi F. Girgis H. Plovanich M. Belcher-Timme C. A. Sancak Y. (2011). Integrative genomics identifies MCU as an essential component of the mitochondrial calcium uniporter. Nat. 476, 341–345. 10.1038/nature10234 21685886 PMC3486726

[B10] Bernardi P. Carraro M. Lippe G. (2022). The mitochondrial permeability transition: recent progress and open questions. FEBS J. 289, 7051–7074. 10.1111/febs.16254 34710270 PMC9787756

[B11] Bogenhagen D. (2012). Mitochondrial DNA nucleoid structure. Biochim. Biophys. Acta 1819, 914–920. 10.1016/j.bbagrm.2011.11.005 22142616

[B12] Borcherding N. Brestoff J. R. (2023). The power and potential of mitochondria transfer. Nature 623, 283–291. 10.1038/s41586-023-06537-z 37938702 PMC11590279

[B13] Brown T. Tkachuk A. Shtengel G. Kopek B. Bogenhagen D. Hess H. (2011). Superresolution fluorescence imaging of mitochondrial nucleoids reveals their spatial range, limits, and membrane interaction. Mol. Cell Biol. 31, 4994–5010. 10.1128/MCB.05694-11 22006021 PMC3233019

[B14] Burman J. Pickles S. Wang C. Sekine S. Vargas J. N. S. Zhang Z. (2017). Mitochondrial fission facilitates the selective mitophagy of protein aggregates. J. Cell Biol. 216, 3231–3247. 10.1083/jcb.201612106 28893839 PMC5626535

[B15] Caicedo J. Cooper S. Heigwer F. Warchal S. Qiu P. Molnar C. (2017). Data-analysis strategies for image-based cell profiling. Nat. Methods 14, 849–863. 10.1038/nmeth.4397 28858338 PMC6871000

[B16] Calì T. Ottolini D. Negro A. Brini M. (2012). α-Synuclein controls mitochondrial calcium homeostasis by enhancing endoplasmic reticulum-mitochondria interactions. J. Biol. Chem. 287, 17914–17929. 10.1074/jbc.M111.302794 22453917 PMC3365710

[B17] Calvo-Rodriguez M. Hou S. Snyder A. Kharitonova E. K. Russ A. N. Das S. (2020). Increased mitochondrial calcium levels associated with neuronal death in a mouse model of Alzheimer’s disease. Nat. Commun. 11, 2146. 10.1038/s41467-020-16074-2 32358564 PMC7195480

[B18] Carpenter A. Jones T. Lamprecht M. Clarke C. Kang I. H. Friman O. (2006). CellProfiler: image analysis software for identifying and quantifying cell phenotypes. Genome Biol. 7, R100. 10.1186/gb-2006-7-10-r100 17076895 PMC1794559

[B19] Chakrabarti R. Ji W. Stan R. de Juan Sanz J. Ryan T. Higgs H. (2018). INF2-mediated actin polymerization at the ER stimulates mitochondrial calcium uptake, inner membrane constriction, and division. J. Cell Biol. 217, 251–268. 10.1083/jcb.201709111 29142021 PMC5748994

[B20] Chen B. Legant W. Wang K. Shao L. Milkie D. E. Davidson M. W. (2014). Lattice light-sheet microscopy: imaging molecules to embryos at high spatiotemporal resolution. Sci. 346, 1257998. 10.1126/science.1257998 25342811 PMC4336192

[B21] Cho B. Cho H. Jo Y. Kim H. D. Song M. Moon C. (2017). Constriction of the mitochondrial inner compartment is a priming event for mitochondrial division. Nat. Commun. 8, 15754. 10.1038/ncomms15754 28598422 PMC5472732

[B22] Cogliati S. Frezza C. Soriano M. Varanita T. Quintana-Cabrera R. Corrado M. (2013). Mitochondrial cristae shape determines respiratory chain supercomplexes assembly and respiratory efficiency. Cell 155, 160–171. 10.1016/j.cell.2013.08.032 24055366 PMC3790458

[B23] Colom A. Derivery E. Soleimanpour S. Tomba C. Molin M. D. Sakai N. (2018). A fluorescent membrane tension probe. Nat. Chem. 10, 1118–1125. 10.1038/s41557-018-0127-3 30150727 PMC6197433

[B24] Csordás G. Várnai P. Golenár T. Roy S. Purkins G. Schneider T. G. (2010). Imaging interorganelle contacts and local calcium dynamics at the ER-mitochondrial interface. Mol. Cell 39, 121–132. 10.1016/j.molcel.2010.06.029 20603080 PMC3178184

[B25] De Stefani D. Raffaello A. Teardo E. Szabò I. Rizzuto R. (2011). A forty-kilodalton protein of the inner membrane is the mitochondrial calcium uniporter. Nat. 476, 336–340. 10.1038/nature10230 21685888 PMC4141877

[B26] Dong L. Kovarova J. Bajzikova M. Bezawork-Geleta A. Svec D. Endaya B. (2017). Horizontal transfer of whole mitochondria restores tumorigenic potential in mitochondrial DNA-Deficient cancer cells. eLife 6, e22187. 10.7554/eLife.22187 28195532 PMC5367896

[B27] Du H. Guo L. Fang F. Chen D. Sosunov A. A. McKhann G. M. (2008). Cyclophilin D deficiency attenuates mitochondrial and neuronal perturbation and ameliorates learning and memory in Alzheimer’s disease. Nat. Med. 14, 1097–1105. 10.1038/nm.1868 18806802 PMC2789841

[B28] D’Alessandro M. Kanaan S. Geller M. Praticò D. Daher J. (2025). Mitochondrial dysfunction in Alzheimer’s disease. Ageing Res. Rev. 107, 102713. 10.1016/j.arr.2025.102713 40023293

[B29] Fang E. Hou Y. Palikaras K. Adriaanse B. A. Kerr J. S. Yang B. (2019). Mitophagy inhibits amyloid-β and tau pathology and reverses cognitive deficits in models of Alzheimer’s disease. Nat. Neurosci. 22, 401–412. 10.1038/s41593-018-0332-9 30742114 PMC6693625

[B30] Figley M. Gu W. Nanson J. Shi Y. Sasaki Y. Cunnea K. (2021). SARM1 is a metabolic sensor activated by an increased NMN/NAD^+^ ratio to trigger axon degeneration. Neuron 109, 1118–1136. 10.1016/j.neuron.2021.02.009 33657413 PMC8174188

[B31] Franco A. Dang X. Zhang L. Molinoff P. Dorn G. W. 2nd (2022). Mitochondrial dysfunction and pharmacodynamics of mitofusin activation in murine Charcot-Marie-Tooth disease type 2A. J. Pharmacol. Exp. Ther. 383, 137–148. 10.1124/jpet.122.001332 36507849 PMC9553116

[B32] Frezza C. Cipolat S. Martins de Brito O. Micaroni M. Beznoussenko G. V. Rudka T. (2006). OPA1 controls apoptotic cristae remodeling independently from mitochondrial fusion. Cell 126, 177–189. 10.1016/j.cell.2006.06.025 16839885

[B33] Friedman J. Lackner L. West M. DiBenedetto J. Nunnari J. Voeltz G. (2011). ER tubules mark sites of mitochondrial division. Sci. 334, 358–362. 10.1126/science.1207385 21885730 PMC3366560

[B34] Fry M. Navarro P. Hakim P. Ananda V. Qin X. Landoni J. (2024). *In situ* architecture of Opa1-dependent mitochondrial cristae remodeling. EMBO J. 43, 391–413. 10.1038/s44318-024-00027-2 38225406 PMC10897290

[B35] Gao Y. Spahn C. Heilemann M. Kenney L. (2018). The pearling transition provides evidence of force-driven endosomal tubulation during salmonella infection. mBio 9. e01083-18. 10.1128/mBio.01083-18 29921673 PMC6016247

[B36] Garrido N. Griparic L. Jokitalo E. Wartiovaara J. van der Bliek A. Spelbrink J. (2003). Composition and dynamics of human mitochondrial nucleoids. Mol. Biol. Cell 14, 1583–1596. 10.1091/mbc.E02-07-0399 12686611 PMC153124

[B37] Geisler S. Holmström K. Skujat D. Fiesel F. C. Rothfuss O. C. Kahle P. J. (2010). PINK1/Parkin-mediated mitophagy is dependent on VDAC1 and p62/SQSTM1. Nat. Cell Biol. 12, 119–131. 10.1038/ncb2012 20098416

[B38] Giacomello M. Pyakurel A. Glytsou C. Scorrano L. (2020). The cell biology of mitochondrial membrane dynamics. Nat. Rev. Mol. Cell Biol. 21, 204–224. 10.1038/s41580-020-0210-7 32071438

[B39] Giorgi C. Missiroli S. Patergnani S. Duszynski J. Wieckowski M. Pinton P. (2015). Mitochondria-associated membranes: composition, molecular mechanisms, and physiopathological implications. Antioxid. Redox Signal 22, 995–1019. 10.1089/ars.2014.6223 25557408

[B40] Goldstein R. Nelson P. Powers T. Seifert U. (1996). Front propagation in the pearling instability of tubular vesicles. J. Phys. II France 6, 767–796. 10.1051/jp2:1996210

[B41] Griswold J. Bonilla-Quintana M. Pepper R. Lee C. T. Raychaudhuri S. Ma S. (2025). Membrane mechanics dictate axonal pearls-on-a-string morphology and function. Nat. Neurosci. 28, 49–61. 10.1038/s41593-024-01813-1 PMC11706780 39623218

[B42] Gu Y. Zhao X. Zhang N. Yang Y. Yi Y. Shao Q. (2024). Mitochondrial dysfunction as a therapeutic strategy for neurodegenerative diseases: current insights and future directions. Ageing Res. Rev. 102, 102577. 10.1016/j.arr.2024.102577 39528070

[B43] Guan F. Wu X. Zhou J. Lin Y. He Y. Fan C. (2024). Mitochondrial transfer in tunneling nanotubes-a new target for cancer therapy. J. Exp. Clin. Cancer Res. 43, 147. 10.1186/s13046-024-03069-w 38769583 PMC11106947

[B44] Gwosch K. Pape J. Balzarotti F. Hoess P. Ellenberg J. Ries J. (2020). MINFLUX nanoscopy delivers 3D multicolor nanometer resolution in cells. Nat. Methods 17, 217–224. 10.1038/s41592-019-0688-0 31932776

[B45] Hayakawa K. Esposito E. Wang X. Terasaki Y. Liu Y. Xing C. (2016). Transfer of mitochondria from astrocytes to neurons after stroke. Nature 535, 551–555. 10.1038/nature18928 27466127 PMC4968589

[B46] Ishihara T. Ban-Ishihara R. Ota A. Ishihara N. (2022). Mitochondrial nucleoid trafficking regulated by the inner-membrane AAA-ATPase ATAD3A modulates respiratory complex formation. PNAS 119, e2210730119. 10.1073/pnas.2210730119 PMC9704698 36383603

[B47] Islam M. N. Das S. R. Emin M. T. Wei M. Sun L. Westphalen K. (2012). Mitochondrial transfer from bone-marrow-derived stromal cells to pulmonary alveoli protects against acute lung injury. Nat. Med. 18, 759–765. 10.1038/nm.2736 22504485 PMC3727429

[B48] Jiao H. Jiang D. Hu X. Du W. Ji L. Yang Y. (2021). Mitocytosis, a migrasome-mediated mitochondrial quality-control process. Cell 184, 2896–2910. 10.1016/j.cell.2021.04.027 34048705

[B49] Kazi N. Klink N. Gallant K. Kipka G. Gersch M. (2025). Chimeric deubiquitinase engineering reveals structural basis for specific inhibition of the mitophagy regulator USP30. Nat. Struct. Mol. Biol. 32, 1776–1786. 10.1038/s41594-025-01534-4 40325251 PMC12440824

[B50] Korobova F. Ramabhadran V. Higgs H. (2013). An actin-dependent step in mitochondrial fission mediated by the ER-associated formin INF2. Sci. 339, 464–467. 10.1126/science.1228360 23349293 PMC3843506

[B51] Kostic M. Ludtmann M. Bading H. Hershfinkel M. Steer E. Chu C. T. (2015). PKA phosphorylation of NCLX reverses mitochondrial calcium overload and depolarization, promoting survival of PINK1-deficient dopaminergic neurons. Cell Rep. 13, 376–386. 10.1016/j.celrep.2015.08.079 26440884 PMC4709126

[B52] Kukat C. Wurm C. Spåhr H. Falkenberg M. Larsson N. Jakobs S. (2011). Super-resolution microscopy reveals that Mammalian mitochondrial nucleoids have a uniform size and frequently contain a single copy of mtDNA. PNAS 108, 13534–13539. 10.1073/pnas.1109263108 21808029 PMC3158146

[B53] Kukat C. Davies K. Wurm C. Spåhr H. Bonekamp N. A. Kühl I. (2015). Cross-strand binding of TFAM to a single mtDNA molecule forms the mitochondrial nucleoid. PNAS 112, 11288–11293. 10.1073/pnas.1512131112 26305956 PMC4568684

[B54] Landoni J. C. Lycas M. D. Macuada J. Stepp W. Jaccard R. Obara C. J. (2026). Pearling drives mitochondrial DNA nucleoid distribution. Science 392, 102–109. 10.1126/science.adu5646 41926598

[B55] Lazarou M. Sliter D. Kane L. Sarraf S. A. Wang C. Burman J. L. (2015). The ubiquitin kinase PINK1 recruits autophagy receptors to induce mitophagy. Nat. 524, 309–314. 10.1038/nature14893 26266977 PMC5018156

[B56] Léon C. Brassard-Jollive N. Gonzalez-Rodriguez D. Riveline D. (2025). Tube into pearls: a membrane-driven pearling instability shapes platelet biogenesis. Mol. Biol. Cell 36, rt1. 10.1091/mbc.E25-01-0045 40802286 PMC12586887

[B57] Lewis M. Lewis W. (1915). Mitochondria (and other cytoplasmic structures) in tissue cultures. Am. J. Anat. 17, 339–401. 10.1002/aja.1000170304

[B58] Lewis S. Uchiyama L. Nunnari J. (2016). ER-mitochondria contacts couple mtDNA synthesis with mitochondrial division in human cells. Sci. 353, aaf5549. 10.1126/science.aaf5549 27418514 PMC5554545

[B59] Liu H. Mao H. Ouyang X. Lu R. Li L. (2024a). Intercellular mitochondrial transfer: the novel therapeutic mechanism for diseases. Traffic 25, e12951. 10.1111/tra.12951 39238078

[B60] Liu H. Xie J. Zhen C. Zeng L. Fan H. Zhuang H. (2024b). Nucleoid-phagy: a novel safeguard against mitochondrial DNA-Induced inflammation. Autophagy 20, 2821–2823. 10.1080/15548627.2024.2395145 PMC11587825 39225184

[B61] López-Otín C. Blasco M. Partridge L. Serrano M. Kroemer G. (2023). Hallmarks of aging: an expanding universe. Cell 186, 243–278. 10.1016/j.cell.2022.11.001 36599349

[B62] Macuada J. Molina-Riquelme I. Vidal G. Pérez-Bravo N. Vásquez-Trincado C. Aedo G. (2024). OPA1 and disease-causing mutants perturb mitochondrial nucleoid distribution. Cell Death Dis. 15, 870. 10.1038/s41419-024-07165-9 39616197 PMC11608329

[B63] Mageswaran S. K. Grotjahn D. A. Zeng X. Barad B. A. Medina M. Hoang M. H. (2023). Nanoscale details of mitochondrial constriction revealed by cryoelectron tomography. Biophysical J. 122, 3768–3782. 10.1016/j.bpj.2023.07.030 PMC10541493 37533259

[B64] Mahamid J. Pfeffer S. Schaffer M. Villa E. Danev R. Cuellar L. K. (2016). Visualizing the molecular sociology at the HeLa cell nuclear periphery. Sci. 351, 969–972. 10.1126/science.aad8857 26917770

[B65] McWilliams T. Prescott A. Allen G. Tamjar J. Munson M. J. Thomson C. (2016). mito-QC illuminates mitophagy and mitochondrial architecture *in vivo* . J. Cell Biol. 214, 333–345. 10.1083/jcb.201603039 27458135 PMC4970326

[B66] Narendra D. Tanaka A. Suen D. Youle R. (2008). Parkin is recruited selectively to impaired mitochondria and promotes their autophagy. J. Cell Biol. 183, 795–803. 10.1083/jcb.200809125 19029340 PMC2592826

[B67] Narendra D. Jin S. Tanaka A. Suen D. F. Gautier C. A. Shen J. (2010). PINK1 is selectively stabilized on impaired mitochondria to activate parkin. PLoS Biol. 8, e1000298. 10.1371/journal.pbio.1000298 20126261 PMC2811155

[B68] Nelson P. Powers T. Seifert U. (1995). Dynamical theory of the pearling instability in cylindrical vesicles. Phys. Rev. Lett. 74, 3384–3387. 10.1103/PhysRevLett.74.3384 10058187

[B69] Newman L. Weiser Novak S. Rojas G. Tadepalle N. Schiavon C. R. Grotjahn D. A. (2024). Mitochondrial DNA replication stress triggers a pro-inflammatory endosomal pathway of nucleoid disposal. Nat. Cell Biol. 26, 194–206. 10.1038/s41556-023-01343-1 PMC11026068 38332353

[B70] Osterloh J. Yang J. Rooney T. Fox A. N. Adalbert R. Powell E. H. (2012). dSarm/Sarm1 is required for activation of an injury-induced axon death pathway. Sci. 337, 481–484. 10.1126/science.1223899 22678360 PMC5225956

[B71] Palese F. Rakotobe M. Zurzolo C. (2025). Transforming the concept of connectivity: unveiling tunneling nanotube biology and their roles in brain development and neurodegeneration. Physiol. Rev. 105, 1823–1865. 10.1152/physrev.00023.2024 40067081

[B72] Palty R. Silverman W. Hershfinkel M. Caporale T. Sensi S. L. Parnis J. (2010). NCLX is an essential component of mitochondrial Na^+^/Ca^2+^ exchange. PNAS 107, 436–441. 10.1073/pnas.0908099107 20018762 PMC2806722

[B73] Pasquier J. Guerrouahen B. Al Thawadi H. Ghiabi P. Maleki M. Abu-Kaoud N. (2013). Preferential transfer of mitochondria from endothelial to cancer cells through tunneling nanotubes modulates chemoresistance. J. Transl. Med. 11, 94. 10.1186/1479-5876-11-94 23574623 PMC3668949

[B74] Patron M. Checchetto V. Raffaello A. Teardo E. Vecellio Reane D. Mantoan M. (2014). MICU1 and MICU2 finely tune the mitochondrial Ca^2+^ uniporter by exerting opposite effects on MCU activity. Mol. Cell 53, 726–737. 10.1016/j.molcel.2014.01.013 24560927 PMC3988891

[B75] Phinney D. Di Giuseppe M. Njah J. Sala E. Shiva S. St Croix C. M. (2015). Mesenchymal stem cells use extracellular vesicles to outsource mitophagy and shuttle microRNAs. Nat. Commun. 6, 8472. 10.1038/ncomms9472 26442449 PMC4598952

[B76] Pickrell A. Youle R. (2015). The roles of PINK1, parkin, and mitochondrial fidelity in Parkinson’s disease. Neuron 85, 257–273. 10.1016/j.neuron.2014.12.007 25611507 PMC4764997

[B77] Plateau J. (1873). Statique Expérimentale Et Théorique Des Liquides Soumis Aux Seules Forces Moléculaires. Paris: Gauthier-Villars.

[B78] Qin J. Guo Y. Xue B. Shi P. Chen Y. Su Q. P. (2020). ER-mitochondria contacts promote mtDNA nucleoids active transportation via mitochondrial dynamic tubulation. Nat. Commun. 11, 4471. 10.1038/s41467-020-18202-4 32901010 PMC7478960

[B79] Quintanilla R. Jin Y. von Bernhardi R. Johnson G. (2013). Mitochondrial permeability transition pore induces mitochondria injury in huntington disease. Mol. Neurodegener. 8, 45. 10.1186/1750-1326-8-45 24330821 PMC3878840

[B80] Rayleigh L. (1878). On the instability of jets. Proc. Lond Math. Soc. s1-10, 4–13. 10.1112/plms/s1-10.1.4

[B81] Rizea R. Corlatescu A. Costin H. Dumitru A. Ciurea A. (2024). Understanding amyotrophic lateral sclerosis: pathophysiology, diagnosis, and therapeutic advances. Int. J. Mol. Sci. 25, 9966. 10.3390/ijms25189966 39337454 PMC11432652

[B82] Rizzuto R. De Stefani D. Raffaello A. Mammucari C. (2012). Mitochondria as sensors and regulators of calcium signalling. Nat. Rev. Mol. Cell Biol. 13, 566–578. 10.1038/nrm3412 22850819

[B83] Rose K. Herrmann E. Kakudji E. Lizarrondo J. Celebi A. Wilfling F. (2025). *In situ* cryo-ET visualization of mitochondrial depolarization and mitophagic engulfment. Proc. Natl. Acad. Sci. USA 122, e2511890122. 10.1073/pnas.2511890122 PMC12337332 40743392

[B84] Rustom A. Saffrich R. Markovic I. Walther P. Gerdes H.-H. (2004). Nanotubular highways for intercellular organelle transport. Science 303, 1007–1010. 10.1126/science.1093133 14963329

[B85] Saha T. Dash C. Jayabalan R. Khiste S. Kulkarni A. Kurmi K. (2022). Intercellular nanotubes mediate mitochondrial trafficking between cancer and immune cells. Nat. Nanotechnol. 17, 98–106. 10.1038/s41565-021-01000-4 34795441 PMC10071558

[B86] Salvadores N. Sanhueza M. Manque P. Court F. (2017). Axonal degeneration during aging and its functional role in neurodegenerative disorders. Front. Neurosci. 11, 451. 10.3389/fnins.2017.00451 28928628 PMC5591337

[B87] Schermelleh L. Ferrand A. Huser T. Eggeling C. Sauer M. Biehlmaier O. (2019). Super-resolution microscopy demystified. Nat. Cell Biol. 21, 72–84. 10.1038/s41556-018-0251-8 30602772

[B88] Smith E. Shaw P. De Vos K. (2019). The role of mitochondria in amyotrophic lateral sclerosis. Neurosci. Lett. 710, 132933. 10.1016/j.neulet.2017.06.052 28669745

[B89] Soubannier V. McLelland G. Zunino R. Braschi E. Rippstein P. Fon E. A. (2012). A vesicular transport pathway shuttles cargo from mitochondria to lysosomes. Curr. Biol. 22, 135–141. 10.1016/j.cub.2011.11.057 22226745

[B90] Spees J. L. Olson S. D. Whitney M. J. Prockop D. J. (2006). Mitochondrial transfer between cells can rescue aerobic respiration. Proc. Natl. Acad. Sci. U. S. A. 103, 1283–1288. 10.1073/pnas.0510511103 16432190 PMC1345715

[B91] Stringer C. Wang T. Michaelos M. Pachitariu M. (2021). Cellpose: a generalist algorithm for cellular segmentation. Nat. Methods. 18, 100–106. 10.1038/s41592-020-01018-x 33318659

[B92] Sturm G. Hake K. Lefebvre A. Rux C. J. Ivanova D. Millett-Sikking A. (2025). The biophysical mechanism of mitochondrial pearling. Mol. Biol. Cell ar142, ar142. 10.1091/mbc.E25-06-0302 41060791 PMC12586885

[B93] Sun N. Yun J. Liu J. Malide D. Liu C. Rovira I. I. (2015). Measuring *in vivo* mitophagy. Mol. Cell 60, 685–696. 10.1016/j.molcel.2015.10.009 26549682 PMC4656081

[B94] Sun N. Youle R. Finkel T. (2016). The mitochondrial basis of aging. Mol. Cell 61, 654–666. 10.1016/j.molcel.2016.01.028 26942670 PMC4779179

[B95] Tábara L. Segawa M. Prudent J. (2025). Molecular mechanisms of mitochondrial dynamics. Nat. Rev. Mol. Cell Biol. 26, 123–146. 10.1038/s41580-024-00785-1 39420231

[B96] Torralba D. Baixauli F. Sánchez-Madrid F. (2016). Mitochondria know no boundaries: mechanisms and functions of intercellular mitochondrial transfer. Front. Cell Dev. Biol. 4, 107. 10.3389/fcell.2016.00107 27734015 PMC5039171

[B97] Twig G. Elorza A. Molina A. Mohamed H. Wikstrom J. D. Walzer G. (2008). Fission and selective fusion govern mitochondrial segregation and elimination by autophagy. EMBO J. 27, 433–446. 10.1038/sj.emboj.7601963 18200046 PMC2234339

[B98] Ulshöfer R. Bros H. Hauser A. Niesner R. Paul F. Malla B. (2022). Preventing axonal sodium overload or mitochondrial calcium uptake protects axonal mitochondria from oxidative stress-induced alterations. Oxid. Med. Cell Longev. 2022, 6125711. 10.1155/2022/6125711 35663200 PMC9157283

[B99] van den Ameele J. Prudent J. (2026). Mitochondrial genomes on a string of pearls. Sci. 392, 26–28. 10.1126/science.aeg3426 41926602

[B100] Wang X. Su B. Lee H. Li X. Perry G. Smith M. (2009). Impaired balance of mitochondrial fission and fusion in Alzheimer’s disease. J. Neurosci. 29, 9090–9103. 10.1523/JNEUROSCI.1357-09.2009 19605646 PMC2735241

[B101] Wang X. Winter D. Ashrafi G. Schlehe J. Wong Y. L. Selkoe D. (2011). PINK1 and parkin target miro for phosphorylation and degradation to arrest mitochondrial motility. Cell 147, 893–906. 10.1016/j.cell.2011.10.018 22078885 PMC3261796

[B102] Webster H. deF. (1962). Transient, focal accumulation of axonal mitochondria during the early stages of Wallerian degeneration. J. Cell Biol. 12, 361–383. 10.1083/jcb.12.2.361 14005486 PMC2106025

[B103] Wolf S. Mutsafi Y. Dadosh T. Ilani T. Lansky Z. Horowitz B. (2017). 3D visualization of mitochondrial solid-phase calcium stores in whole cells. eLife 6, e29929. 10.7554/eLife.29929 29106371 PMC5703638

[B104] Yamashita S. Jin X. Furukawa K. Hamasaki M. Nezu A. Otera H. (2016). Mitochondrial division occurs concurrently with autophagosome formation but independently of Drp1 during mitophagy. J. Cell Biol. 215, 649–665. 10.1083/jcb.201605093 27903607 PMC5147001

[B105] Yang C. Svitkina T. M. (2019). Ultrastructure and dynamics of the actin-myosin II cytoskeleton during mitochondrial fission. Nat. Cell Biol. 21, 603–613. 10.1038/s41556-019-0313-6 30988424 PMC6499663

[B106] Yu Z. Wang H. Tang W. Wang S. Tian X. Zhu Y. (2021). Mitochondrial Ca^2+^ oscillation induces mitophagy initiation through the PINK1-Parkin pathway. Cell Death Dis. 12, 632. 10.1038/s41419-021-03913-3 PMC8214625 34148057

[B107] Zanfardino P. Longo G. Amati A. Morani F. Picardi E. Girolamo F. (2023). Mitofusin 2 mutation drives cell proliferation in charcot-marie-tooth 2A fibroblasts. Hum. Mol. Genet. 32, 333–350. 10.1093/hmg/ddac201 35994048

[B108] Zhang L. Trushin S. Christensen T. Bachmeier B. V. Gateno B. Schroeder A. (2016). Altered brain energetics induces mitochondrial fission arrest in Alzheimer’s disease. Sci. Rep. 6, 18725. 10.1038/srep18725 26729583 PMC4700525

[B109] Zhao Y. Sun X. Hu D. Prosdocimo D. A. Hoppel C. Jain M. K. (2019). ATAD3A oligomerization causes neurodegeneration by coupling mitochondrial fragmentation and bioenergetics defects. Nat. Commun. 10, 1371. 10.1038/s41467-019-09291-x 30914652 PMC6435701

